# Unraveling the layers of epigenetic control in respiratory antiviral defense

**DOI:** 10.1128/jvi.01783-25

**Published:** 2026-05-20

**Authors:** Matthew I. McFadden, Bryan Simo, Adriana Forero

**Affiliations:** 1 Department of Microbial Infection and Immunity, Infectious Diseases Institute, College of Medicine, The Ohio State University Wexner Medical Center 12306, Columbus, Ohio, USA; Indiana University Bloomington, Bloomington, Indiana, USA

**Keywords:** interferons, epigenetics, host response, respiratory pathogens

## Abstract

The host defense against emerging respiratory pathogens begins with the induction of cell-intrinsic, interferon-mediated antiviral responses. The interferon response induces innate effector and adaptive cellular responses crucial for viral clearance and the establishment of long-lasting immune memory. Although these antiviral processes are primarily characterized at the transcriptional level, the epigenetic mechanisms that orchestrate the cellular transcriptional output during infection remain understudied. Technological advances in systems immunology and virology have revealed dynamic changes in the cellular epigenetic landscape following infection, and their contextual roles in the fine-tuning of antiviral defense. This minireview covers our current understanding of how DNA methylation, post-translational modifications of histones, and chromatin remodelers are dynamically reprogrammed during respiratory virus infections, and the distinct strategies that respiratory viruses employ to subvert epigenetic control. We place further emphasis on cell-type-specific programs and the biological factors that alter the epigenetic landscape and regulate the balance between protective or pathogenic immune responses to infection.

## INTRODUCTION

The host interferon (IFN) response is the first line of defense against viral infection. The recognition of viral nucleic acids by pattern recognition receptors (PRR) activates IFN regulatory factor 3 (IRF3) and nuclear factor kappa-B (NF-kB) to mediate virus stress inducible genes and cell-intrinsic type I (IFNα/β) and III (IFNλ) IFN gene expression (reviewed in references 1, 2). IFNs engage cognate receptors to activate Signal Transducer and Activator of Transcription 1 (STAT1), STAT2, and IRF9 (ISGF3 transcriptional complex) to stimulate IFN-stimulated gene (ISG) transcription (1). ISGs encode antiviral effectors and proinflammatory cytokines that recruit immune cells to sites of infection. This makes the induction of a robust IFN response crucial in bridging cell-intrinsic antiviral immunity with long-lasting adaptive immune responses. The IFN response is fundamentally controlled at the level of transcription (3), underscoring the importance of understanding how epigenetic modifications to DNA and histones that alter chromatin accessibility and structure can serve as an additional antiviral regulatory layer (4).

While canonical IFN signaling pathways and their antagonism by respiratory pathogens have been extensively characterized (5–8), much less is understood about how epigenetically regulated predetermined states and dynamic changes in the host chromatin landscape arise and resolve throughout infection to influence IFN responsiveness, viral clearance, and disease outcomes. Inflammatory cues have been well associated with induced changes in chromatin architecture to support the expression of effector genes. Non-surprisingly, viruses have evolved distinct strategies to antagonize epigenetic programs, subverting host innate immune responses and sustaining effective genome replication. This is best understood in chronic infections with DNA viruses, such as herpesviruses and human immunodeficiency virus, where viral latency and reactivation are controlled by the host epigenetic machinery (reviewed in references 9–15). The interface of epigenetic regulation of host responses to acute respiratory viral infection remains nascent. To date, techniques that capture rapid global changes in chromatin accessibility and protein composition have revealed that epigenetic reprogramming is a conserved consequence of infection across respiratory pathogens (reviewed in reference 9). Current efforts focus on defining how host inflammatory signaling establishes transient and durable chromatin states, as well as the distinct strategies viruses employ to subvert this control.

Here, we will discuss the major epigenetic mechanisms that have been revealed to shape host antiviral responses. We focus on how dynamic DNA methylation, histone modifications, and chromatin accessibility influence IFN signaling and immune memory following infection. Next, our discussion will extend to our current understanding of strategies by which major respiratory viruses, including orthomyxoviruses, pneumoviruses, coronaviruses, and adenoviruses, exploit the epigenetic machinery to subvert host immunity. Finally, we provide perspectives on how host biological factors, including immune history, genetic variation, age, and sex, impart epigenetic control over antiviral responses and contribute to the heterogeneity in respiratory viral disease outcomes across populations. A better understanding of virus-host epigenetic interactions will facilitate rational, context-dependent antiviral interventions.

## MECHANISMS OF HOST EPIGENETIC REGULATION

### DNA methylation

**Fig 1:**
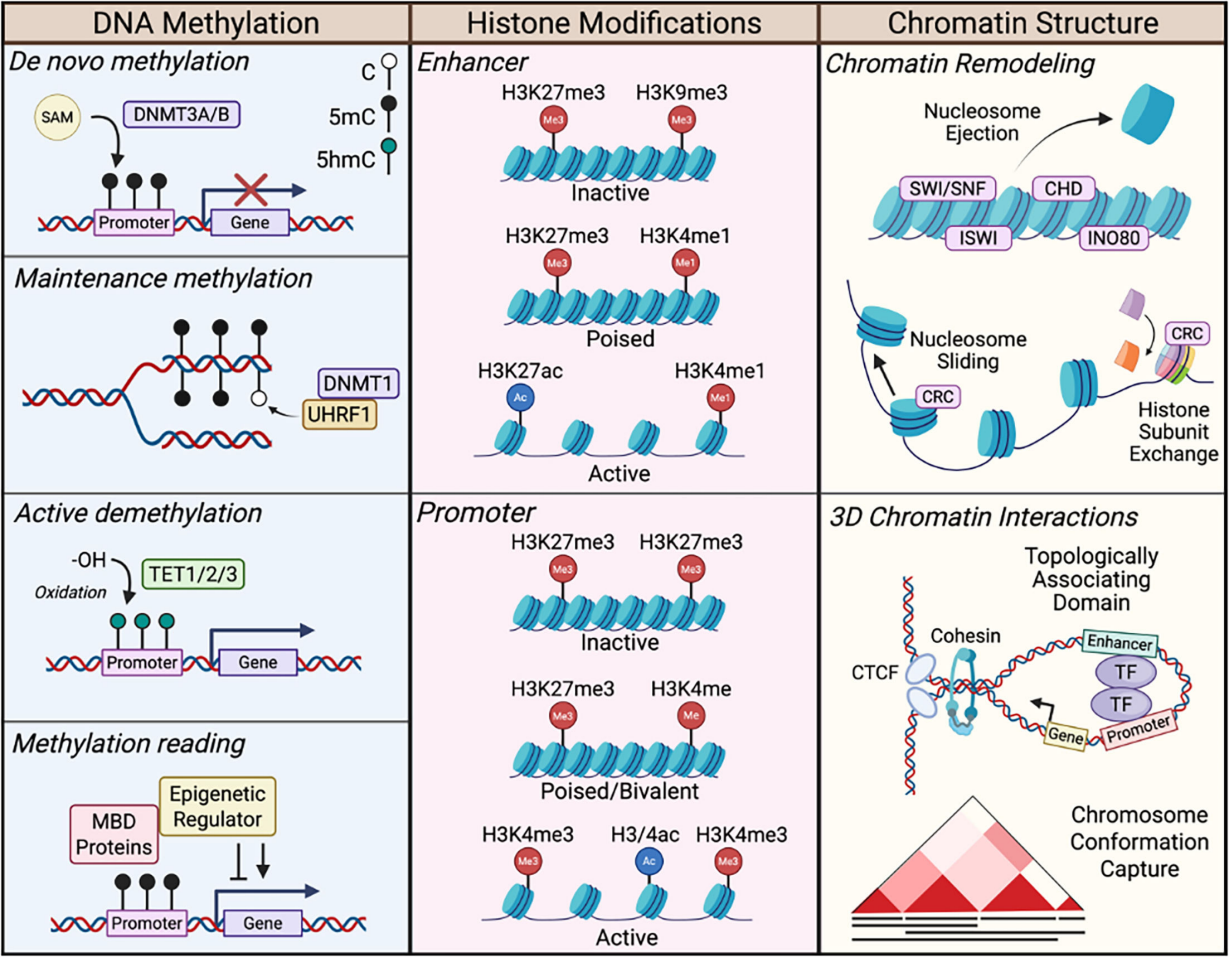
Overview of epigenetic regulatory mechanisms. Epigenetic regulation through (left) DNA methylation, (center) histone modifications, and (right) chromatin dictates transcriptional responses. (Left) *De novo* DNA methylation at CpG sites is performed by DNA methyltransferase 3A (DNMT3A) and DNMT3B, resulting in 5-methylcytosine (5mC). Ubiquitin-like with PHD and Ring Finger Domains 1 (UHRF1) recruits DNMT1 to hemi-methylated cytosine (C) bases during genome replication to maintain heritable DNA methylation patterns. TET enzymes oxidize 5mC to 5-hydroxymethylcytosine (5hmC) and its derivatives to perform active demethylation. 5mC and 5hmC are recognized by MBD proteins that interact with epigenetic remodelers and transcriptional regulators, positively or negatively regulating transcription. (Center) Histone modifications by histone acetyltransferases (HAT), deacetylases (HDAC), and methyltransferases occur at enhancer and promoter regulatory sites. The methylation (Me) and acetylation (Ac) of lysines shape local chromatin structure to silence (inactive) or enable (active) transcription. Poised/bivalent loci contain permissive and repressive histone modifications, holding chromatin in an inaccessible state that can be rapidly remodeled upon stimulation to allow for transcription of stimulus-responsive genes. (Right) Chromatin remodeler complexes (CRCs) restructure nucleosomes through ejection, repositioning (sliding), or exchanging canonical subunits for more specialized histone variants (top). Chromatin interacts to form three-dimensional (3D) structures, defining regulatory regions where enhancers, promoters, and transcription factors (TF) interact to drive transcription. These regions are termed topologically associating domains (TADs) and are often demarcated by CCCTC-binding factor (CTCF) and cohesin. Chromosome Conformation Capture (3C) techniques enable mapping of these 3D interactions, driving recent advances in understanding epigenetic regulation of host responses to viral infection (bottom). Created in BioRender.

DNA methyltransferase (DNMT) family enzymes catalyze the addition of a methyl (–CH_3_) group to the 5-carbon of cytosine (C) residues, converting it to 5-methylcytosine (5-mC), often at CpG dinucleotides (16). DNA methylation is established *de novo* by DNMT3A and DNMT3B and maintained during DNA replication by Ubiquitin-like with PHD and Ring Finger Domains 1 (UHRF1)-mediated recruitment of DNMT1 to hemi-methylated DNA (Fig. 1, left). 5-mC is generally associated with transcriptional repression due to steric hindrance of transcription factor binding to DNA response elements, or through recognition of these marks by methyl-CpG-binding domain (MBD)-containing proteins that promote heterochromatin (17) (Fig. 1, left). Demethylation can occur passively through loss of DNMT1-mediated maintenance, or actively through ten eleven translocation (TET) enzyme-mediated oxidation of 5-mC to 5-hydroxymethylcytosine (5-hmC), itself a stable epigenetic mark, and further derivatives, followed by base-excision repair (16). This allows for dynamic regulation of methylation-coordinated transcriptional programs that define cellular identity and response to stimuli (Fig. 1, left).

Genome-wide DNA methylation profiling approaches have established DNA methylation as a central regulator of immunity (Table 1) (reviewed in reference 18). Some sequencing technologies rely on sodium bisulfite-mediated deamination of unmethylated cytosines, converting them to uracil while 5-mC residues remain unchanged, to monitor changes in the methylome. Whole-genome bisulfite sequencing (WGBS) monitors thymine substitutions to provide single-base pair resolution of DNA methylation patterns across the genome. Reduced representation bisulfite sequencing (RRBS) uses methylation-insensitive restriction enzymes to enrich for CpG-rich regions of DNA. This approach cost-effectively increases coverage depth at these regulatory regions (19, 20). Other sequencing-based techniques rely on protein-based enrichment techniques that use either antibodies raised against 5-mC or 5-hmC (methylated DNA immunoprecipitation sequencing; MeDIP-seq), or recombinant MBD protein domains (MBD-seq), to capture methylation-rich regions (21–23). While these methods provide computationally and cost-efficient genome-wide coverage of cytosine methylation, they lack the single-base-pair resolution of bisulfite sequencing. The advent of long-read sequencing technologies has eliminated the need for bisulfite conversion, allowing direct and simultaneous measurement of DNA modifications, such as 5-mC or 5-hmC, in a single experiment (reviewed in reference 24). To reduce both the cost and the computational burden associated with whole-genome sequencing, DNA methylation microarrays capture bisulfite-converted DNA using probes targeting predefined CpG sites. These platforms enable methylome studies across large cohorts (25, 26).

**TABLE 1:** Epigenetic mapping techniques

Regulatory level	Method	Description
DNA methylation	Whole-genome bisulfite sequencing (WGBS)	Sodium bisulfite converts unmethylated cytosines to uracil while leaving 5-mC unchanged, enabling single-base resolution, genome-wide methylation profiling (18, 19)
	Reduced representation bisulfite sequencing (RRBS)	Restriction enzyme enrichment of CpG-dense regions prior to bisulfite sequencing, increasing sensitivity (18, 20)
	Methylated DNA immunoprecipitation sequencing (MeDIP-seq)	Antibody-based 5-mC or 5-hmC enrichment prior to sequencing; this method lacks single-base resolution (21, 22)
	Methyl-CpG-binding domain protein sequencing (MBD-seq)	MBD proteins selectively bind CpG-methylated DNA to enrich methylated fragments for sequencing; this approach lacks single-base resolution (23)
	Microarray	Methylation microarrays combine bisulfite conversion with probes targeting CpG sites at known regulatory regions, enabling large-scale profiling of relevant sites while reducing costs compared with sequencing-based methods (25, 26)
Histone modifications	Chromatin immunoprecipitation (ChIP)	Antibodies isolate DNA bound by proteins or modified histones, followed by mapping through quantitative PCR (ChIP-qPCR) or sequencing (ChIP-seq) (27, 28)
	Cleavage under targets and release using nuclease (CUT&RUN)	Antibodies targeting histone PTMs or chromatin-associated proteins recruit a protein A-micrococcal nuclease (Mnase) fusion protein for targeted cleavage of DNA and sequencing; this method requires less input and provides higher signal-to-noise than ChIP (29, 30)
	Cleavage under targets and tagmentation (CUT&Tag)	Antibodies targeting histone PTMs or chromatin-associated proteins recruit a protein A-Tn5 transposase to simultaneously fragment DNA and insert sequencing adapters at accessible sites (31)
Chromatin organization and remodeling	Micrococcal nuclease sequencing (MNase-seq)	MNase digestion of genomic DNA cleaves exposed linker DNA while sparing nucleosome-associated DNA, mapping nucleosome occupancy (32, 33)
	DNase I hypersensitivity sites sequencing (DNase-seq)	DNase I preferentially cleaves accessible chromatin, and the resulting fragments are sequenced to identify DNase hypersensitive sites to map genome-wide open regulatory elements (32, 34)
	Assay for transposase accessible chromatin using sequencing (ATAC-seq)	A hyperactive Tn5 transposase fragments accessible chromatin and inserts sequencing adapters to map open regulatory elements genome-wide using low input material (32, 35, 36)
	Hi-C	Crosslinked chromatin is digested and proximity-ligated to join interacting DNA fragments, which are sequenced to map genome-wide chromatin interactions to define three-dimensional genome organization (37, 38)

Together, these methods have been used to show that precise methylation patterns guide normal cellular development and function. Disruption in DNA methylation patterns is linked to immunodeficiencies and cancer predisposition (39), conditions associated with heightened risk of infections. Viral infections themselves have been associated with the remodeling of DNA methylation at immune regulatory loci, with viral disease severity driving the magnitude of these changes. This is not merely a marker of infection, but rather the interplay between changes induced by the host response to infection and epigenetic perturbation by viral genes. A critical gap in our understanding is how pre-existing and infection-induced dynamic reprogramming of methylation landscapes shapes the trajectory of acute viral infections and immune memory toward future exposures.

### Histone modifications

Histone subunits undergo post-translational modifications (PTMs) that impact the structure of chromatin. The major PTMs that alter chromatin accessibility and recruitment of remodelers and transcriptional regulators are histone acetylation (Ac) and methylation (Me). These modifications are repeatedly engaged during immune activation and viral infection and set the timing, magnitude, and durability of host transcriptional responses. The acetylation of histones is catalyzed by histone acetyltransferases (HATs) that transfer acetyl groups (CH_3_CO–) from acetyl CoA to the ɛ-amino group of lysine (K) residues on histone tails. This neutralizes the positive charge on histones, weakening interactions with negatively charged DNA. Histone acetylation is recognized by bromodomain (BRD)-containing proteins that nucleate the assembly of chromatin-remodeling complexes that drive gene activation (40, 41). Removal of acetyl groups by histone deacetylases (HDACs) favors heterochromatin formation and transcriptional repression (40). Lysine methyltransferases carry out histone methylation, which, unlike acetylation, does not alter the electrostatic interaction between DNA and histones. Instead, methylated lysine residues serve as recognition sites for epigenetic and transcriptional regulator proteins, bidirectionally regulating transcription. In particular, H3K4me3 is associated with transcriptional activation, and H3K27me3 and H3K9me3 are associated with gene silencing (40, 41). Histone PTMs are particularly important at enhancer and promoter regions, defining the basal and inducible repressive or permissive chromatin states that control transcription (42) (Fig. 1, middle).

While western blotting and mass spectrometry are important tools for identifying and quantifying bulk histone PTMs (43), the ability to map targeted loci has been instrumental in defining histone PTMs as an active determinant of immune gene regulation (Table 1). Chromatin immunoprecipitation paired with polymerase chain reaction or next-generation sequencing (ChIP-PCR or ChIP-seq) has long been the standard method for mapping histone PTMs at target regions (27, 28). Cleavage under targets and release using nuclease (CUT&RUN) and its derivative, CUT and tagmentation (CUT&TAG), are more recent strategies that pair micrococcal nuclease (MNase) or Tn5 transposase, fused to protein A, to identify and cleave protein-specific antibody-bound genomic regions. These methods have improved mapping resolution and dramatically reduced the required cellular input, enabling the identification of regulatory histone PTM in less abundant and primary cell populations, uncovering infection-induced kinetic changes in activating and repressive histone marks across cell types (29–31, 44). These studies demonstrate that both host- and virus-associated mechanisms alter histone PTMs to either attenuate or potentiate inflammatory responses. The identification of virus-encoded histone mimics (45) and other proteins that engage host chromatin regulators has driven an interest in understanding the intersection between host epigenetics and successful viral replication and immune evasion. These insights have motivated the repurposing of existing pharmacologic modulators of chromatin-modifying enzymes as tractable targets to improve host resilience to respiratory viruses and disease outcomes (46).

### Chromatin organization and remodeling

Chromatin remodeling complexes work in tandem with histone PTMs to dynamically regulate chromatin structure and accessibility. These ATP-dependent remodeler complexes, which include the SWI/SNF, ISWI, CHD, and INO80 families (reviewed in references 47, 48), recognize specific histone modifications and interrupt DNA-histone interactions to eject, reposition, or exchange histone subunits in nucleosomes (Fig. 1, right). Nucleosome positioning and spacing can be measured using micrococcal nuclease sequencing (MNase-seq), in which MNase cleaves exposed linker DNA while sparing nucleosome-associated DNA (32, 33). Chromatin regions that are accessible to transcription factors and machinery can be mapped by DNase I hypersensitivity assays (DNase-seq) (34) or the assay for transposase-accessible chromatin using sequencing (ATAC-seq) (35, 36), allowing researchers to capture dynamic changes in accessible chromatin during immune insults and tissue repair (Table 1) (reviewed in reference 32). *In vivo* application of these approaches to infected lung tissue has been most tractable for ATAC-seq, particularly with the advent of single-cell and multi-“omic” strategies. When integrated with transcription factor binding motif and occupancy analyses, these methods reveal cell-type-specific and disease-specific chromatin accessibility patterns that govern transcriptional responses in the infected lung. The accessibility of chromatin at antiviral gene loci is critical for the timely activation of IFN and inflammatory transcriptional programs (49). This is hijacked by viruses to redistribute host nucleosomes and chromatin remodelers onto viral genomes, establishing transcriptionally favorable chromatin states that promote genome maintenance and evade host immune detection (11, 12, 14, 50).

Beyond local chromatin accessibility, higher-order genome organization further shapes gene regulation. The genome is partitioned into active euchromatin and inactive heterochromatin, as reflected by patterns of short- and long-range chromatin interactions that are organized into topologically associating domains (TADs) and cohesin-mediated CTCF loops. These structures restrict enhancer-promoter communication and enforce gene regulatory specificity (51, 52) (Fig. 1, right). Chromosome Conformation Capture (3C)-based approaches involve crosslinking chromatin, followed by restriction enzyme digestion and proximity ligation to generate DNA fragments that encode spatial chromatin contacts, enabling the mapping of higher-order chromatin structure (reviewed in reference 53). These methods, including high-throughput variants like Hi-C (Table 1) (37, 38), have revealed infection-induced alterations in three-dimensional (3D) chromatin architecture that remodel immune gene networks and influence the magnitude, specificity, and durability of host antiviral responses in airway cells (54, 55) (Fig. 1, right).

### RNA modifications and non-coding RNA

While our emphasis is on DNA- and chromatin-level regulation of host-pathogen interactions, epitranscriptomic mechanisms also contribute to the fine-tuning of antiviral immunity. Modifications of host and viral RNA influence the recognition of microbe-associated molecular patterns, innate immune activation, and vaccine efficacy. These mechanisms have been elegantly summarized in recent reviews (56). Unbiased sequencing technologies like methylated RNA immunoprecipitation, followed by sequencing (MeRIP-seq) and Nanopore direct RNA sequencing, have demonstrated that respiratory viruses across multiple families hijack the host RNA methyltransferase machinery to promote N^6^-methyladenosine (m^6^A) modification of viral RNA as a replicative and immune-evasion strategy (57–61). In parallel, virus-encoded long non-coding RNAs (lncRNAs) and host lncRNAs induced after PRR activation and IFN signaling (62) function as microRNA “sinks” and alter chromatin accessibility (63) to regulate the host response to viral infection (reviewed in references 64–66). Collectively, these findings underscore the RNA-based epigenetic regulatory tier that complements chromatin-level control of respiratory antiviral immunity.

## EPIGENETIC REGULATION OF HOST ANTIVIRAL RESPONSES

### Regulation of viral recognition and cell-intrinsic IFN responses

Epigenetic heterogeneity underlies the cell-type specificity of IFN production (67). IFN stimulation further induces an epigenetic reprogramming, both acute and heritable, at a subset of ISG loci (68). This supports the notion that prior inflammatory exposures alter cell-type-specific chromatin landscapes to imprint transcriptional “memory” that dictates the magnitude and durability of future antiviral responses. Promoters, generally found near transcription start sites, directly recruit RNA polymerase II (Pol II) through transcription factor binding. In contrast, distal enhancer elements exist in distinct functional states (i.e., poised, active, or latent) that determine their responsiveness to signaling cues. Poised enhancers are pre-marked by histone H3 mono-methylation (H3K4me1) but lack activating acetylation marks, whereas latent enhancers are unmarked and inaccessible at baseline but acquire activation marks like H3K4me1 and H3K27ac (Fig. 1, center) (69). These enhancer states are established by cell-type-restricted, lineage-defining pioneer factors, permitting rapid activation by stimulus-responsive transcription factors (i.e., IRF and STATs) at select IFN and ISG loci to expand the antiviral transcriptional program, as well as recruited cofactors that sustain robust gene transcription (70, 71). Consistent with this model, ATAC-seq and Hi-C experiments show that IFN stimulation triggers rapid chromatin remodeling at ISG loci (54, 72). These structural changes correlate with the deposition of activating histone marks (H3K27ac) and the recruitment of Pol II, linking enhancer activation to higher-order chromatin organization.

Histone acetylation is a central and unexpectedly permissive feature of IFN-stimulated transcription. HDAC activity is required for efficient ISGF3-mediated transactivation of ISGs in response to IFN treatment (73–76). One study proposed that HDAC1 mediates this activity, although a second study was unable to validate this association, likely due to cell-type-specific differences across studies or to compensatory upregulation of other HDACs following HDAC1 knockdown (73, 74). Notably, components of HDAC-associated corepressor complexes also influence IFN responses. Silencing of Switch-independent 3A (SIN3A) disrupts the HDAC1/2-containing Sin3a complex and reduces basal ISG expression, impairing IFNα-mediated antiviral protection against influenza A virus (IAV) (77). These findings indicate that HDAC activity maintains chromatin in a poised, signal-responsive state that enables ISGF3 engagement and productive transcription rather than enforcing static repression. As a result, HDAC inhibitors render A549 human airway epithelial cells susceptible to infection by IAV and human metapneumovirus (HMPV) (74). However, the role of HDAC in regulating intrinsic immunity against viral infections is more complex than the positive regulation of IFN responses. In other viral models, such as herpesviruses, HDAC inhibition can both enhance host defense mechanisms that limit infectivity and derepress viral gene expression (78–80). These discrepancies suggest that modulating HDAC activity to tip the balance toward host protection is largely dependent on both viral life cycles and the tissues they target (46).

Readers of acetylated histone marks, including several Bromodomain and Extra-Terminal domain (BET) proteins (BRD2, BRD3, BRD4, BRDT), coordinate IFN-induced transcription. BRD2 cooperates with the histone acetyltransferase KAT2A to evict the regulatory histone variant H2A.Z, facilitating ISGF3 recruitment to ISG promoters (81). BRD4 promotes rapid ISG induction by recruiting Positive Transcription Elongation Factor b (P-TEFb) to release Pol II (82). ChIP-seq demonstrated that P-TEFb recruitment depends on HDAC activity, partially explaining its paradoxical requirement for positive regulation of antiviral responses (83). However, BET proteins can also exert context-dependent suppressive effects on IFN responses. BRD4 stabilizes the chromatin regulator SUPT16H in association with HDAC1 and EZH2, dampening ISG transcription (84). The physiological relevance of BET proteins in antiviral immunity is emphasized by *in vitro* and *in vivo* SARS-CoV-2 infection models. BET inhibition suppresses virus-induced IFN responses, leading to increased viral replication in the lung and worsened disease outcomes (85). Similarly, pan-BET inhibition increases mortality following influenza infection (86), collectively suggesting that BET proteins are instrumental in translating histone PTM remodeling at antiviral loci into an effective transcriptional response.

ATP-dependent chromatin remodelers of the SWI/SNF family (i.e., canonical BAF, PBAF, and non-canonical BAF complexes) also dictate host IFN responses. These complexes mobilize and evict nucleosomes to establish accessible promoter and enhancer states that permit IRF/ISGF3 engagement with target sequences. Biotin ligation assays showed that BRD9, a subunit of the non-canonical BAF complex, interacts with STAT2 and co-binds ISG promoters to facilitate transcription and mediate antiviral protection against IAV infection *in vitro* (87). Similarly, the SWI/SNF component ARID1A is recruited by IRF3 to promote permissive chromatin states at type I IFN gene promoters in murine macrophages (88). As such, SWI/SNF remodelers integrate HDAC- and BET-driven mechanisms to tune chromatin accessibility, enhancer activation, and the kinetics of IFN/ISG induction during antiviral responses.

As opposed to histone-mediated regulation of ISG transcription downstream of IFN signaling, DNA methylation is primarily known to control the expression of type I IFN genes. In contrast with the typical repressive function of DNA methylation, DNMT3A stabilizes CpG methylation upstream of *Hdac9*, inhibiting the association of the histone methyltransferase Enhancer of Zeste Homolog 2 (EZH2) and deposition of repressive H3K27me3 at the *Hdac9* promoter. In turn, HDAC9 interacts with TANK-binding kinase 1 (TBK1) via its deacetylase domain, enhancing TBK1 activity and promoting IRF3 phosphorylation to drive IFN gene expression (89). On the other hand, UHRF1 and DNMT1 can restrict IFN responses by maintaining CpG methylation upstream of *Ifnb1,* thereby inhibiting IRF3 recruitment (90). Targeted demethylation of a single cytosine residue is sufficient to restore *Ifnb1* expression and antiviral responses. This observation exemplifies how small changes in this regulatory layer can have significant effects at the cellular, tissue, or organismal levels (90). Furthermore, differential methylation of IFN genes has been associated with autoimmune conditions (91), defining this as a broad innate immune regulatory mechanism across pathologies. How pre-existing or induced DNA methylation patterns shape cell-type-specific antiviral responses, and their conservation across pathogens, remains to be explored in depth.

Transcription factors that recruit chromatin modifiers can also define the subtypes of IFN that are expressed. Zinc finger E-box binding homeobox 1 (ZEB1) guides HDACs and DNMTs to target promoters and selectively represses IFNλ1 following dsRNA stimulation (92). Additionally, TGFβ-dependent upregulation of ZEB1 (93) represses IRF1 and heightens susceptibility to respiratory syncytial virus (RSV) and rhinovirus infection (94, 95). Broader chromatin features likewise influence IFN responses. Preexisting chromatin states determine STAT1/STAT2 occupancy, enabling cell-type-specific responses to IFN (96). IFN exposure itself imprints trained immunity, marked by shifts in histone modifications (68, 96). Overall, IFN stimulation remodels ISG-dense chromatin regions, increasing accessibility, enhancer activation, and chromatin contacts to shape ISG expression (54). Together, these studies provide foundational evidence of the multilayered epigenetic mechanisms that define the kinetics, amplitude, and cell-type specificity of cell-intrinsic innate antiviral transcriptional programs.

### Regulation of cellular innate and adaptive immunity

The identity of myeloid and innate lymphoid cells (ILCs) is highly dictated by epigenetic imprinting (reviewed in references 42, 97–101). These patterns are altered in response to viral infections, resulting in lasting epigenetic changes or “training” of these cells. SARS-CoV-2 infection drives chromatin remodeling of IFN, ISG, and inflammatory genes in alveolar macrophages (AMs), which potentiate antiviral responses against secondary IAV infections (102). Similar cross-pathogen protection resulting from trained immunity has been observed after remodeling of monocyte-derived AM following IAV infection (103). These studies demonstrate the strength of imprinting following a singular viral infection, so it remains to be seen how subsequent exposures to seasonal respiratory viruses can further enforce this reprogramming. Similar chromatin remodeling at antiviral genes in the myeloid compartment was observed in an IAV-challenged human cohort, suggesting that mechanisms of trained immunity may be shared across species (104). When comparing healthy and SARS-CoV-2 convalescent individuals, peripheral blood mononuclear cells exhibit well-defined trajectories of maturation and activation as defined by single-cell chromatin accessibility profiling and show durable epigenetic imprinting associated with recovery from infection (105). Of note, SARS-CoV-2 infection alters the epigenetic landscape of hematopoietic stem and progenitor cells in both mice and humans, suggesting that these alterations are not unique to terminally differentiated cells and could result in a permanent shift in future responses to viral challenge across hematopoietic lineages (106).

ILCs encompass the natural killer (NK) cells, ILC1s, ILC2s, and ILC3s and function as rapid effectors of lung antiviral and repair responses. Like myeloid cells, ILCs have also been shown to undergo training following viral infection (98, 99). Murine cytomegalovirus-trained NK cells preferentially expand and exhibit enhancement in both cytokine and cytotoxic responses to secondary viral exposure (107). In contrast, murine memory-like NK cells induced by IAV infection have diminished cytotoxicity (108) and enhanced cytokine responses (108, 109) upon secondary IAV exposure. Human IAV vaccination induces the emergence of a memory NK cell population capable of increased cytokine production upon secondary IAV antigen exposure, mirroring findings in mice, but this population appears transient (110, 111). Given that IAV peptide-specific NK cell clones have been observed in human cohorts, it is reasonable to assume that these memory populations are long-lived. Whether the ILC training elicited by vaccination mirrors that induced by a bona fide IAV infection remains unknown (112). Recent evidence suggests that ILC1s, ILC2s, and ILC3s also undergo immune training following IAV infection, but the epigenetic underpinnings of these observations and their consequences to respiratory viral disease remain poorly defined (113). Collectively, these studies demonstrate that IAV infection and vaccination drive ILC training and reveal a need to better understand its durability and relevance to other respiratory viral infections.

T and B lymphocytes are essential antiviral effector cells with highly coordinated development, activation, and memory programs. The function of these cells is largely epigenetically regulated (reviewed in references 114–119). Across respiratory viruses, antigenic priming leaves durable epigenetic imprints that shape the quality of T cell memory. Antigen-specific cytotoxic CD8+ T cells are reprogrammed through rapid modification of histone methylation at promoters and enhancers during IAV infection (40). In naïve CD8+ T cells, bivalent promoters, marked by permissive H3K4me3 and repressive H3K27me3, maintain silencing of activation-associated gene programs. Following activation, these regions rapidly lose H3K27me3, permitting transcription of these genes (120). Effector loci are primed by “poised” enhancers, marked by H3K4 mono- or dimethylation and H3K27me3, that gain H3K27ac during activation (121). In naïve T cells, H3K4me3 marks active enhancers upstream of loci associated with stemness, while in effector and memory T cells, H3K4me3 is redistributed to enhancers upstream of activation- and function-associated loci (121). Similar chromatin changes are seen in SARS-CoV-2 S-specific CD4+ T cells following infection. Interestingly, infection results in greater accessibility of ISG loci, enhanced cytotoxicity, and decreased proliferation relative to S-specific CD4+ T cells induced by vaccination. This suggests that inflammatory signals associated with bona fide infection influence recall responses at the chromatin level (122, 123). Additionally, DNA methylation dictates T cell fate following IAV infection, as impairment of *de novo* DNA methylation and maintenance DNA methylation inhibit effector CD8+ T cell differentiation and regulatory CD4+ T cell function, respectively (124, 125). Thus, multiple epigenetic regulatory layers work in concert to coordinate T cell activation, effector responses, memory formation, and tissue repair during respiratory viral infection, and a deeper understanding of these complex interactions will enable more precise control of T cell-directed therapies.

In B cells, the magnitude and quality of recall responses are dictated by epigenetic remodelers. In particular, EZH2 regulates histone PTMs and chromatin accessibility to coordinate transcriptional programs that control B cell fate determination during infection (126, 127). Following IAV infection, EZH2 catalyzes H3K27me3 deposition at BLIMP1 target genes, promoting the formation of effector antibody-secreting cells. EZH2 deletion results in reduced serum antibody titers and diminished humoral responses to IAV (127, 128). The H3K79 methyltransferase DOT1L is also essential for germinal center formation and memory responses following IAV infection, but specific target loci were not identified (129). Consistent with these histone modification-driven programs, chromatin accessibility provides a functional readout of memory B cell quality. T-bet-expressing B cells, predictive of long-lasting vaccine-induced humoral immunity, show increased accessibility at transcription factor loci associated with the regulation of immune effector functions (130). Together, these studies demonstrate that epigenetic regulators do not merely support B cell activation but sustain the durability and protective capacity of antiviral humoral immunity.

## VIRAL INTERFERENCE WITH THE HOST EPIGENOME

### Orthomyxoviridae

**Fig 2:**
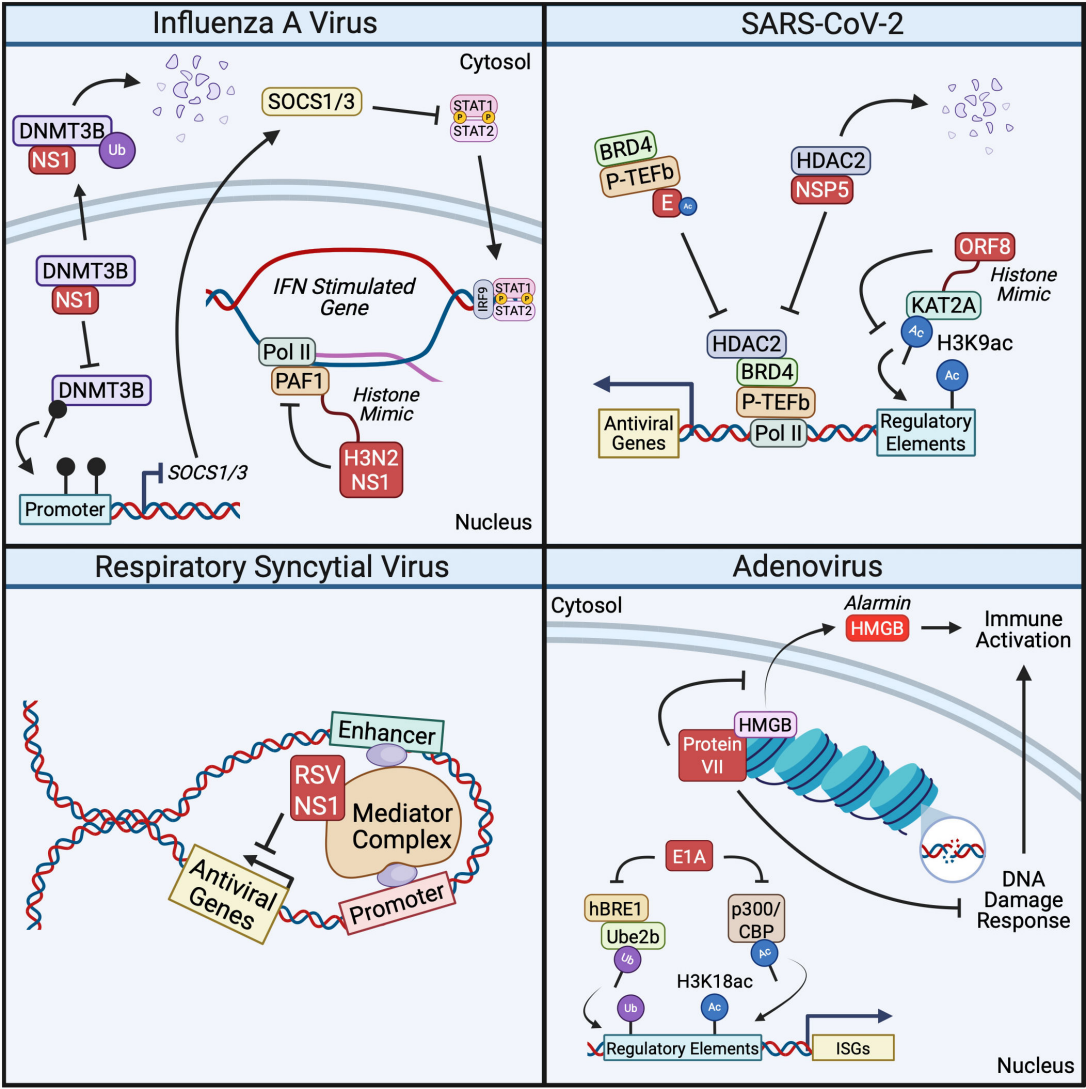
Antagonism of the host antiviral epigenetic response by respiratory viruses. Distinct viral mechanisms of interference with the host epigenome. Influenza A virus (IAV, top left) NS1 facilitates nuclear export and ubiquitin (Ub)-mediated degradation of DNMT3B, to derepress negative regulators of interferon (IFN) signaling (i.e., *SOCS1/3*). The NS1 protein of H3N2 IAV strains is a histone mimic that binds the polymerase-associated factor 1 (PAF1) elongation complex and inhibits RNA polymerase II (Pol II)-mediated transcription. The histone mimic encoded by SARS-CoV-2 (top right), ORF8, binds lysine acetyltransferase 2A (KAT2A), inhibiting H3K9ac marks at antiviral genes. Bromodomain-containing protein 4 (BRD4) promotes transcription downstream of inflammatory signals by recruiting Positive Transcription Elongation Factor b (P-TEFb), which releases Pol II, in a histone deacetylase (HDAC)-dependent manner. SARS-CoV-2 NSP5 binds and mediates degradation of HDAC2, and its E protein binds BRD4, inhibiting its recruitment to acetylated lysine residues. Respiratory syncytial virus (bottom left) inhibits antiviral gene transcription by binding to the Mediator complex. Adenovirus (bottom right) E1A protein inhibits activating H3K18ac deposition by p300/CREB-binding protein (CBP) and H2B monoubiquitination by hBRE1 and Ube2b. Protein VII binds high-mobility group box (HMGB) proteins to sequester them in chromatin and prevent their release as alarmins, and it inhibits DNA damage responses that drive immune activation. Created in BioRender.

The Orthomyxoviridae family, which includes influenza A and B viruses, comprises segmented negative-sense RNA viruses that suppress antiviral transcription and modulate innate immune signaling through interactions with host nuclear and chromatin-associated proteins (Table 2). Among respiratory viruses, IAV provides one of the clearest examples of epigenetic immune antagonism mediated by a single viral protein. The IAV H3N2 non-structural protein 1 (NS1) is a polyfunctional protein that encodes an N-terminal RNA-binding domain and a C-terminal effector domain that harbors an ARSK motif, which mimics histone H3 and promotes antiviral evasion (131) and viral replication (132, 133). Post-translational modifications of the NS1 histone-mimic motif facilitate interactions with chromatin readers like Chromodomain-Helicase DNA-binding 1 (CHD1) and Polymerase-Associated Factor 1 (PAF1) at actively transcribed antiviral genes (134). Global Run-On sequencing (GRO-seq), a genomic technique used to measure nascent RNA, shows that viruses unable to bind PAF1 fail to repress Pol II elongation across antiviral genes (Fig. 2) (134). Together, these data establish H3N2 NS1 as a functional viral histone mimic that counteracts host antiviral responses by targeting transcriptional elongation rather than initiation. Importantly, this histone mimic is not conserved in NS1 proteins of other IAV strains, suggesting alternative NS1-mediated antiviral antagonism mechanisms are sufficient to suppress host responses and permit viral replication.

**TABLE 2:** Epigenetic mechanisms of respiratory virus interference with host defense

Family	Virus	Viral protein	Host cellular target	Regulatory level	Mechanism
Orthomyxoviridae	IAV (H1N1, H3N2)	NS1	DNMT3B	DNA methylation	NS1-mediated degradation of DNMT3B derepresses negative regulators of JAK-STAT signaling (135)
	IAV (H3N2)	NS1	Nucleolin	DNA methylation	NS1 sequesters nucleolin to promote hypermethylation at rRNA promoters (136)
	IAV (H3N2)	NS1	PAF1	Transcription elongation	H3N2 uniquely encodes a histone mimic motif in its NS1 protein that binds PAF1 to inhibit transcription elongation at antiviral genes (134)
	IAV (H5N1)	NS1	Undefined	Histone modifications	NS1 from highly pathogenic H5N1 IAV mediates loss of permissive H3K4me3 and gain of repressive H3K27me3 at ISG promoters (137)
	IAV (H5N1)	NS1	Undefined	DNA methylation; histone modification	NS1 induces H3K4me3 at promoters of antigen presentation genes, suppressing their expression (138)
Pneumoviridae	RSV	Undefined	KDM5B and KDM6	Histone modifications	Infection induces upregulation of the H3K4 demethylase *Kdm5b* and the H3K27 demethylase *Kdm6* in dendritic cells, balancing type I IFN and pro-inflammatory cytokine production (139–141)
	RSV	Undefined	BRD4	Histone modifications	During infection, BRD4 promotes NF-κB-dependent pro-inflammatory gene expression and mediates H3K122 acetylation (142)
	RSV	NS1	MED25	Chromatin organization	RSV NS1 interacts with the Mediator complex subunit, MED25, to inhibit antiviral gene expression (143–145)
	HMPV	M2-2	Undefined	Histone modifications	M2-2 mediates dysregulation of histone subunit expression and inhibits global H3K27me3 in HMPV-infected A549 cells (146)
Coronaviridae	SARS-CoV-2	ORF8	KAT2A	Histone modifications	The histone mimic contained within ORF8 binds and reduces protein levels of the H3K9 acetyltransferase KAT2A, inhibiting deposition of permissive H3K9ac at antiviral genes (147)
	SARS-CoV-2	E	BRD2/4	Histone modification reader	Acetylated E protein binds the second bromodomain of BRD4 to inhibit IFN production in A549 and Calu3 cells, while E interaction with the SEED domain of BRD2/4 promotes ISG expression in HEK293T cells (147–149)
	SARS-CoV-2	NSP5	HDAC2	Histone modifications	NSP5 cleaves HDAC2, inhibiting deacetylase activity and dampening ISG expression (148, 150)
Adenoviridae	Adenovirus	E1A	p300/CBP	Histone modifications	E1A inhibits the HAT p300/CBP to prevent activation of transcription factors and deposition of activating H3K18ac (151–153)
	Adenovirus	E1A	hBRE1/RNF20	Histone modifications	E1A blocks recruitment of the E2 conjugase Ube2b to E3 ubiquitin ligase hBRE1/RNF20, preventing H2B monoubiquitination and suppressing ISG expression (154)
	Adenovirus	Protein VII	Nucleosomes	Chromatin organization	Protein VII contains a histone mimic motif that facilitates interactions with host nucleosomes to alter DNA accessibility and inhibit DNA damage responses (155, 156)
	Adenovirus	Protein VII	HMGB1	Chromatin organization	Protein VII, through its interaction with host nucleosomes, sequesters HMGB1 to prevent its release into extracellular space, where it acts as an alarmin to initiate cellular immune responses (155, 157)
	Adenovirus	E4orf3	PML	Nuclear structure	E4orf3 causes reorganization of the nuclear scaffold PML, inhibiting ISG expression (158)
	Adenovirus (Ad5)	E4orf3	Mre11	Nuclear structure	Ad5 E4orf3 uniquely causes restructuring of the Mre11 DNA repair complex to prevent its antagonism of adenovirus replication (159)
Parvoviridae	HBoV	NS1	DNMT1	DNA methylation	NS1 promotes the degradation of DNMT1 to prevent methylation of the viral genome, which inhibits viral RNA processing (160)

Both H1N1- and H3N2-encoded NS1 inhibit IFN signal transduction by relocalizing DNMT3B to the cytoplasm and promoting its K48-linked ubiquitination-dependent proteasomal degradation (135). This relieves promoter methylation of negative regulators of JAK-STAT signaling (e.g., SOCS1/3, PIAS1/3, PTP1B, TCPTP), inducing their expression and attenuating IFN signal transduction (Fig. 2) (135). Beyond its epigenetic interference with Pol II-driven mRNA transcription, H3N2 NS1 also interferes with ribosomal RNA (rRNA) synthesis. H3N2 NS1 sequesters nucleolin from rRNA promoters, driving promoter hypermethylation and transcriptional silencing of rRNA (136). In epithelial cells, where IFN responses depend on rapid transcriptional and translational output (3), repression of rRNA synthesis may further constrain host antiviral immunity (136).

Comparative studies have revealed strain-specific differences in NS1-driven epigenetic remodeling that shape disease outcomes. In highly pathogenic H5N1 infection, but not in pandemic H1N1 infection, a subset of ISGs is repressed via changes in histone PTM, including loss of H3K4me3 and gain of H3K27me3 at ISG promoters (137). H5N1 additionally suppresses antigen-presenting pathways through DNA methylation (138). Collectively, these findings support a model in which NS1 deploys non-redundant epigenetic strategies to fine-tune host antiviral responses. These mechanisms are likely to contribute to differences in pathogenicity, immune escape, and clinical outcome, and position NS1-host protein interactions as an axis for potential therapeutic targeting.

### Pneumoviridae (respiratory syncytial virus and human metapneumovirus)

Respiratory syncytial virus (RSV; Orthopneumovirus) and human metapneumovirus (HMPV; Metapneumovirus) are closely related negative-sense RNA viruses that replicate in airway epithelia and induce host epigenetic remodeling during infection. Several human cohort studies indicate that acute RSV infection drives DNA-methylation signatures that correlate with disease severity (161) or sequelae (e.g., recurrent wheezing or asthma) in children (162). Notably, decreased DNA methylation at immune effector loci, such as Perforin 1 (*PRF1*), the cytolytic effector of CD8+ T cells and NK cells, has also been linked to RSV disease susceptibility (163). Together, these studies demonstrate that the host epigenetic landscape both pre-defines disease risk and is dynamically remodeled during infection to dictate infection outcomes.

RSV-induced innate immune activation also drives rapid chromatin remodeling at antiviral promoters and enhancers through PTM of histones, increasing accessibility at ISG loci to enable their expression and combat viral infection while repressing select inflammatory and epithelial-identity elements (Table 2). RSV infection leads to dual upregulation of KDM5 and KDM6 family proteins that demethylate H3K4 and H3K27, respectively, reshaping activating H3K4me3 and repressive H3K27me3 marks to balance immune cell activation and cytokine production (139–141). In parallel, RSV activates BRD4 acetyltransferase activity, which acetylates H3K122 at antiviral and inflammatory gene loci in RSV-infected immortalized human small airway epithelial cells (hSAECs) (142). Interestingly, BRD4 inhibition decreases IFN responses in both hSAEC and RSV-infected mice, increasing viral replication. However, concomitant dampening of virus-induced inflammation reduces airway obstruction and mitigates viral disease (142). This highlights the context-dependent consequences of epigenetic targeting and their contributions to immunopathology across viruses.

Beyond host-driven chromatin remodeling, RSV also encodes viral effectors that directly engage epigenetic regulatory machinery. The RSV non-structural protein 1 (NS1) localizes to the nucleus, associates with chromatin, and interacts with transcriptional co-regulator complexes, including Mediator, to target regulatory elements near antiviral genes and suppress their transcription (Fig. 2) (143–145). This provides a direct viral mechanism for shaping host transcriptional responses, complementing host-mediated histone and DNA-methylation changes induced during infection. Together, these findings indicate that RSV reshapes the host epigenetic landscape at multiple levels, coupling immediate histone- and chromatin-based modulation of innate immunity with longer-term DNA-methylation programs that may influence immune memory and susceptibility to subsequent disease. Like RSV, HMPV infection induces dynamic remodeling of repressive H3K27 methylation at innate immune loci. The HMPV M2-2 PDZ-binding motif regulates histone subunit expression and inhibits H3K27 methylation (146). However, the genomic loci impacted by altered H3K27 methylation and its impact on gene expression remain unresolved, marring the biological significance of these changes in the context of HMPV disease outcomes.

### Coronaviridae (SARS-CoV-2, SARS-CoV, MERS-CoV, endemic HCoVs)

Since the onset of the COVID-19 pandemic, a growing body of research has revealed large-scale epigenome remodeling resulting from SARS-CoV-2 infection. Infection leads to widespread changes in DNA methylation, including differentially methylated regions near ISG promoters and inflammatory loci, with patterns more pronounced in severe disease (164). These shifts likely arise from host epigenetic responses rather than direct viral methyltransferase activity, as coronaviruses lack enzymes capable of modifying host DNA. On a larger scale, Hi-C analyses and polymer modeling show that SARS-CoV-2 infection broadly disrupts higher-order genome architecture (44, 165). At ISGs such as *DDX58* and *IFIT*, infection results in a less coherent 3D architecture and reduced enhancer-promoter contact stability, providing a structural explanation for impaired activation of antiviral genes. This reorganization is more pronounced in SARS-CoV-2 than with HCoV-OC43 infection or double-stranded RNA mimic (polyI:C) stimulation, indicating that the severity of inflammation or SARS-CoV-2-specific mechanisms may promote this restructuring (44).

SARS-CoV-2 encodes numerous gene products that antagonize viral recognition and IFN signaling cascades, including viral proteins that directly interact with host chromatin regulators (Table 2) (5). ORF8 contains an ARKS histone H3-mimic motif that becomes acetylated, enabling ORF8 chromatin binding. This interaction drives a widespread shift toward increased repressive histone H3 marks (H3K9me3 and H3K27me3) and decreased activating marks (H3K9ac), dampening the host response to infection (Fig. 2) (147). The envelope (E) protein has also been proposed to drive epigenetic antagonism of IFN responses through competitive binding of BRD2 and BRD4 bromodomains in airway epithelial cells (Fig. 2) (85, 148). Acetylation of conserved lysine residues K53/K63 in E enables the interaction with bromodomains in BRD2/4, representing a potentially shared mechanism of antiviral antagonism between SARS-CoV and SARS-CoV-2 (85). A second study in HEK293T cells confirmed E:BRD2/4 protein interactions but mapped the interactions to the C-terminal intra-virion domain of E and the Ser/Glu/Asp-rich (SEED) C-terminal region of BET proteins. In these cells, E overexpression upregulates ISG expression, suggesting cell-type-specific regulatory mechanisms (149). Lastly, the viral 3CL protease, NSP5, interacts with and cleaves HDAC2, inhibiting ISG induction following IFN stimulation (Fig. 2) (148, 150). NSP5 catalytic sequences are broadly conserved across human coronaviruses, with both alpha- and beta-coronaviruses capable of cleaving HDAC2, albeit with distinct efficiencies. Together, these findings indicate that SARS-CoV-2 leverages epigenetic modulation as a mechanism of IFN antagonism, a strategy partly conserved across coronaviruses and an important target for future mechanistic and therapeutic investigation.

### Adenoviruses (human adenoviruses)

The Adenoviridae, a family of double-stranded DNA viruses, are associated with acute respiratory infections in children and immunocompromised individuals. Successful nuclear viral gene transcription and genome replication require extensive epigenetic reprogramming of the infected cell (Table 2) (reviewed in reference 10). Adenoviruses encode products that can interact with chromatin (E1A and protein VII) and promote nuclear reorganization (E4orf3). These interactions profoundly alter nuclear morphology, chromatin architecture, histone patterns, and host gene expression (166). Early work showed that adenovirus infection is both unaffected by IFN pretreatment and can potently inhibit the antiviral activity of IFN in A549 cells (167). E1A drives some of this inhibition (167, 168), due in part to the inhibition of p300/CBP, which is necessary for the acetylation of transcription factors (151) and the deposition of histone activation marks (H3K18ac) (Fig. 2) (152, 153). E1A associates with the E3 ubiquitin ligase hBRE1/RNF20 to inhibit recruitment of the E2 conjugase Ube2B, preventing monoubiquitination of histone H2B (H2B-ub), a mark of transcriptionally active chromatin that is required for ISG expression (Fig. 2) (154). More recently, it has been shown that protein VII, a histone mimic tethered to incoming viral genomes, binds to host chromatin to alter nucleosome mobility (Fig. 2) (155), suppress antiviral DNA damage responses (156), and immobilize high mobility group box 1 (HMGB1) on chromatin to prevent its release as an alarmin (Fig. 2) (155), ultimately inhibiting the activation of type I IFN responses in human and murine airway cells and cell lines (157). While E4orf3 lacks enzymatic activities that directly reprogram histone modifications, its extensive reorganization of nuclear architecture suppresses IFN and DNA damage response-mediated pathways that antagonize viral genome replication (158, 169, 170). Of note, the E4Orf3 protein function differs across adenovirus species. Reorganization of the antiviral nuclear scaffold PML is broadly conserved, while the restructuring of the DNA repair complex Mre11-Rad50-Nbs1, which restricts viral genome replication, is limited to a subset of species (158, 159). In addition to these factors, adenovirus proteins, like E1B-55K and E4orf6, indirectly influence chromatin-dependent host responses by targeting nuclear restriction and DNA repair complexes for degradation to reinforce a virus-permissive nuclear environment (171). Collectively, these observations raise the possibility that differences among adenovirus subtypes in respiratory tropism and disease severity (172, 173) are influenced, at least in part, by variation in the magnitude and persistence of virus-induced chromatin reprogramming within airway epithelial cells.

### Other respiratory viruses

Picornaviridae (rhinoviruses) and Parvoviridae (human bocavirus [HboV]) constitute major causes of upper and lower respiratory tract infection. These viruses replicate entirely in the cytoplasm and do not possess known histone-mimic motifs or chromatin-binding proteins, as those encoded by influenza and coronaviruses. Therefore, it is likely that the epigenetic changes observed during infection, chromatin remodeling at antiviral loci, or inflammatory DNA methylation shifts in airway mucosa are predominantly driven by the host antiviral response (174–177). Of note, DNMT1-mediated DNA methylation of parvovirus genomes reduces viral gene expression. During HBoV infection, DNMT1 is downregulated by proteasomal degradation mediated by DNMT1 interactions with HBoV non-structural protein 1 (Table 2) (160). Whether this interaction extends beyond viral genome methylation to regulate host DNA methylation patterns and responses to infection remains to be addressed.

## BIOLOGICAL FACTORS THAT SHAPE HOST EPIGENETIC LANDSCAPE AND INFECTION OUTCOMES

Converging evidence supports a unifying model in which respiratory viruses both induce and reshape host epigenetic programs that dictate innate inflammation, immune memory, and tissue repair. The same genome-wide technologies that have enabled the study of chromatin remodeling during infection have been central to identifying alterations in epigenetic signatures associated with biological and environmental factors that render individuals susceptible to viral disease. Genetic lesions in genes encoding epigenetic regulators have been linked to hundreds of developmental disorders termed chromatinopathies (178). The intellectual and developmental delays associated with these disorders underscore the importance of epigenetic maintenance to host homeostasis and tolerance to stress stimuli (179). Interestingly, a subset of individuals with chromatinopathies, with lesions spanning DNA methylation regulators (180–182), histone PTM-modifying enzymes (183–185), and chromatin remodeler proteins (186–188), has increased susceptibility to infection or adverse infection-related outcomes. While much of this risk might stem from secondary symptoms, such as aspiration or dystonia, there is evidence that these genetic lesions may contribute to inborn errors of immunity (189–191). Thus, a more complete understanding of the intersection between epigenetic machinery and antiviral defense has the potential to identify high-risk populations and improve interventions to ameliorate infection outcomes.

Age is another biological factor with a crucial impact on respiratory disease outcomes. The epigenomic immaturity of the immune system contributes to disease severity and the development of asthma following RSV infection in infants (reviewed in reference 192). On the other hand, distinct DNA methylation patterns in aged vs young CD4+ regulatory T cells decrease the efficiency with which these cells support tissue repair following IAV infection (193). Since the onset of the COVID-19 pandemic, various studies have confirmed that SARS-CoV-2 infection leads to long-lasting remodeling of the DNA methylome (194) and have employed machine learning techniques to interrogate their relation to infection status and disease severity (195–199), post-acute sequelae of COVID-19 (200), and epigenetic age, with mixed results (195, 201–205). As these computational approaches continue to be refined, high-confidence associations between epigenetic aging and respiratory viral disease may reliably predict disease risk and enable targeted therapeutic intervention (206). Aging of the lung has also been attributed to a decrease in its regenerative potential and is heavily associated with aberrant DNA methylation (207). Chronic lung diseases, such as chronic obstructive pulmonary disease and idiopathic pulmonary fibrosis, alter the fitness and proliferative potential of lung cells targeted by viruses such as IAV (208). How these epigenetic changes inform the cellular ability to induce crucial biological pathways that dictate the course of infection and repair following viral injury (209) remains to be fully elucidated.

Sex-dimorphic responses to viral infection have been extensively documented (reviewed in references 210, 211). Beyond hormonal and chromosomal-encoded features that shape sex-based differences in the response to infection, sex-specific DNA methylation patterns can also contribute to the establishment of inflammatory responses to infection. Female mice exhibit decreased DNA methylation at the *Tlr7* locus, leading to increased *Tlr7* expression, enhanced germinal center responses, and improved antibody-mediated protection against IAV (212). Whether these distinct epigenetic patterns can be linked back to regulation by sex hormones or chromosomes remains to be established. Lastly, DNA methylation has been at the forefront of regulatory processes associated with age and sex. However, this does not limit the involvement of other chromatin regulators as contributors to varied outcomes across populations.

## CONCLUSIONS AND FUTURE PERSPECTIVES

To date, much of our understanding of epigenetic regulation in development and immunity derives from animal models and model-antigen or vaccination-based systems. These approaches have enabled the identification of epigenetic signatures associated with disease state and risk, informing precision medicine strategies across diverse pathologies (213). However, how these regulatory mechanisms are established, maintained, or perturbed during physiological respiratory viral infection remains an important and largely unresolved question. Emerging technologies that enable targeted manipulation of gene expression and modeling of clinically relevant genetic variance, combined with human-based experimental systems, such as organoids and precision-cut lung slices, provide new opportunities to bridge mechanistic insights from animal models with observational findings from human cohorts, refining the links between epigenetic regulation, infection outcomes, and tissue repair in the respiratory tract. Ultimately, dissecting virus-specific epigenetic dependencies across respiratory viral lifecycles will be essential for distinguishing chromatin-based interventions with targeted or pan-antiviral potential.
